# Distinct Small RNA Signatures in Extracellular Vesicles Derived from Breast Cancer Cell Lines

**DOI:** 10.1371/journal.pone.0161824

**Published:** 2016-08-31

**Authors:** Tonje Fiskaa, Erik Knutsen, Marlen Aas Nikolaisen, Tor Erik Jørgensen, Steinar Daae Johansen, Maria Perander, Ole Morten Seternes

**Affiliations:** 1 Department of Medical Biology, Faculty of Health Sciences, UiT–The Arctic University of Norway, MH-building Breivika, Tromsø, N-9037, Norway; 2 Department of Pharmacy, Faculty of Health Sciences, UiT–The Arctic University of Norway, MH-building Breivika, Tromsø, N-9037, Norway; 3 Marine Genomics group, Faculty of Biosciences and Aquaculture, Nord University, Bodø, Norway; Oxford Brookes University, UNITED KINGDOM

## Abstract

Breast cancer is a heterogeneous disease, and different subtypes of breast cancer show distinct cellular morphology, gene expression, metabolism, motility, proliferation, and metastatic potential. Understanding the molecular features responsible for this heterogeneity is important for correct diagnosis and better treatment strategies. Extracellular vesicles (EVs) and their associated molecules have gained much attention as players in intercellular communication, ability to precondition specific organs for metastatic invasion, and for their potential role as circulating cancer biomarkers. EVs are released from the cells and contain proteins, DNA, and long and small RNA species. Here we show by high-throughput small RNA-sequencing that EVs from nine different breast cancer cell lines share common characteristics in terms of small RNA content that are distinct from their originating cells. Most strikingly, a highly abundant small RNA molecule derived from the nuclear 28S rRNA is vastly enriched in EVs. The miRNA profiles in EVs correlate with the cellular miRNA expression pattern, but with a few exceptions that includes miR-21. This cancer-associated miRNA is retained in breast cancer cell lines. Finally, we report that EVs from breast cancer cell lines cluster together based on their small RNA signature when compared to EVs derived from other cancer cell lines. Altogether, our data demonstrate that breast cancer cell lines manifest a specific small RNA signature in their released EVs. This opens up for further evaluation of EVs as breast cancer biomarkers.

## Introduction

Breast cancer is the most common invasive cancer in women and the leading cause of cancer deaths in females [1]. Importantly, detection of the disease at an early stage significantly increases the 5-year survival rate [2,3]. Therefore, it is of great interest to develop molecular and cellular diagnostic assays with potential to aid early diagnosis, clinical decision-making, and patient management [4].

In the last few years several studies have demonstrated that cancer cells produce and release increased numbers of membranous vesicles into the extracellular environment compared to normal cells [5,6,7,8]. These cancer-derived extracellular vesicles (EVs) carry proteins, DNA, and RNA species from the originating cell [9,10,11,12] and act as mediators of intercellular communication that may influence on the progression of the disease [13,14]. EVs from both cancer cells and associated stromal cells play an important role in altering the tumor environment and may promote tumor cell migration, invasion, and formation of distant metastatic niches [15,16,17,18,19]. EVs have also been demonstrated to play a role in cancer cell immune evasion, suppression of apoptosis, and in the development of drug resistance [20,21,22,23]. Since EVs are detected in all body fluids, including blood, they are increasingly recognized as potential sources for cancer biomarkers [24].

Cancer-derived EVs are in general heterogeneous, but can be divided into two main classes based on their mode of biogenesis and size [25]. These are the exosomes of 30–120 nm that derive from exocytosed multivesicular bodies [26,27,28], and ectosomes that are microvesicles of 120–1000 nm shed from the plasma membrane [29,30]. Numerous reports have shown that EVs derived directly from tumor cells, or from the extracellular fluids of cancer patients, have a distinct molecular signature of proteins [31,32,33], mRNAs [34], and non-coding RNAs [6,35]. In particular, EV-associated micro RNAs (miRNAs) have gained much attention as signaling substances in intercellular communication [36,37,38]. MiRNAs are small non-coding RNAs of approximately 22 nucleotides (nt), which regulate the expression of target genes at the posttranscriptional level. They play key roles in cellular processes like proliferation, differentiation, and survival and are interesting candidates as cancer biomarkers [39,40,41,42]. MiRNA profiling now appears as an important approach in the molecular characterization of tumor subtyping [43], disease progression [44], treatment strategy, and survival [45,46]. Small RNA deep-sequencing have revealed that the cells contain a variety of other small RNA species, and some of them are incorporated into and released in EVs [47,48,49,50]. How RNA species are selected and sorted into EVs have not been identified, but different covalent modifications of miRNAs have been noted that either prevent miRNAs from being incorporated in EVs, or facilitate the incorporation [51]. The functional role of EV-associated small RNAs in cancer progression is still largely unknown.

Here, we use high-throughput sequencing to determine the complete small RNA content in EVs derived from nine breast cancer cell lines. By employing this comprehensive strategy, we have identified common signatures in the breast cancer cell line-derived EVs that are distinct from the intracellular small RNA expression pattern in the originating cells. This difference is mainly due to high contents of non-miRNA small RNAs in EVs, especially a highly abundant small RNA derived from the 28S rRNA. We find, with some exceptions, that the intracellular miRNA expression pattern is mostly reflected in the EVs. Interestingly, the oncogenic miRNA miR-21, which expression is highly associated with breast cancer, is retained in the breast cancer cell lines. This finding supports selectivity in the sorting and secretion of certain miRNA species. Finally, small RNA signatures show that EVs from breast cancer cell lines cluster together, and can be separated from EVs derived from other cancer cell types. This is an important observation that support that small RNAs within cancer-derived EVs may be suitable as breast cancer-specific biomarkers.

## Methods

### Cell culture

Human cancer cell lines MA-11, MDA-MB-231 (ATCC #HTB-26™), Hs578T (ATCC #HTB-126™), AU565 (ATCC #CRL-2351™), HCC1428 (ATCC #CRL-2327™), MCF7 (ATCC #HTB-22™), HCC1187 (ATCC #CRL-2322™), DU4475 (ATCC #HTB-123™), HCC1569 (ATCC #CRL-2330™), DLD1 (ATCC #CCL-221™), HPAF-II (ATCC #CRL-1997™) and LNCaP (ATCC #CRL-1740™), were maintained at 37°C in 5% CO_2_ and cultured in either Dulbecco´s Modified Eagle's Medium (DMEM) or Roswell Park Memorial Institute Medium (RPMI) supplemented with 10% FBS.

All cancer cell lines, except MA-11, were obtained from the American Type Culture Collection (ATCC; www.atcc.org). The human MA-11 breast carcinoma cell line, established from bone marrow micrometastases of a patient with breast cancer [52], was obtained by Øystein Fodstad (Norwegian Radium Hospital, Oslo, Norway). Cell pellets and conditioned media from the Melmet 1 (MM1) and Melmet 5 (MM5) melanoma cell lines were a gift from Dr. Siri Tveito at the Norwegian Radium Hospital, Oslo, Norway, and were established from the biopsies of metastatic melanoma patients at the department of Tumor Biology, The Norwegian Radium Hospital Oslo, Norway [53].

### Extracellular vesicles isolation

EVs were isolated from cell culture media using the Total Exosome Isolation reagent according to the protocol (Invitrogen/Life Technologies), publication number MAN000694. Conditioned media was prepared from approximately 1×10^7^ cells grown at 70% confluency in T175 cell culture flasks. Cell cultures were washed two times using PBS followed by 72 hours incubation with 15 mL exosome-depleted medium at 37°C and 10% CO_2_. Exosome-depleted medium was obtained by subjecting the FBS to ultracentrifugation, 120,000 g for 12 hours, before adding it (10%) to DMEM or RPMI. Conditioned media was harvested and centrifuged for 30 min at 2,000 g and 4°C to remove detached cells and cell debris. The supernatant was transferred to a new tube and 7.5 ml of Total Exosome Isolation reagent was added and the mixture was incubated over night at 2°C. After incubation, the samples were centrifuged for 1h at 10,000 g. The supernatant was discarded and the pellets consisting of extracellular vesicles were resuspended in 200 μl PBS.

### Vesicle size determination

The vesicle size distributions were measured by photon correlation spectroscopy using a Submicron Particle-sizer (Model 360, Nicomp, Santa Barbara, CA, USA). To avoid possible interference caused by dust particles, test tubes were pre-rinsed with distilled water and bath-sonicated for 10 min. In addition, all sample preparations were performed in a laminar airflow bench. The vesicle samples were diluted with filtered (0.2 μm Milipore filters) distilled water to provide appropriate count intensity (approx. 250–350 kHz) and measured in three parallels (run time 10 min at 23°C). Both Gaussian and Nicomp algorithms were fitted to the experimental data to find the distribution that best describes the vesicle population. As the fit error was found to be smaller than 1.5, and the residual error was smaller than 10, Nicomp distribution was selected. The volume-weighted distribution was used to determine the mean diameter and polydispersity index (PI) of all samples.

### Immunoblotting

Cells and isolated EVs were lysed in RIPA buffer (100mM Tris-HCl pH8, 300 mM NaCl, 2% NP-40, 1% sodium deoxycholate, 0.1% SDS) supplemented with Complete Ultra Mini EDTA-free protease inhibitor mixture (1 tablet/10 ml, Roche Applied Science) and HaltTM phosphatase inhibitor cocktail (Thermo Fisher Scientific). 30 μg protein extracts were resolved on a SDS-PAGE gel, transferred to a nitrocellulose membrane, and probed with antibodies recognizing CD63 (sc-5275, Santa Cruz Biotechnology; 0.4 μg/ml) or actin (clone C4, MAB1501, Millipore; 1:500). The blots were developed using IRDye800CW-conjugated goat anti-mouse secondary antibody (926–32210, LI-COR; 1:7000) and the Odyssey IR Imaging system.

### RNA isolation and analysis

Small RNA from EVs was isolated using the Total Exosome RNA and Protein isolation Kit according to the protocol, including “Enriching for Small RNAs” step (Invitrogen/Life Technologies), publication number MAN0006962.

Total RNA from the cultured cells was isolated using the TRIzol reagent (Invitrogen/ Life Technologies) and enriched for small RNAs using the PureLink™ miRNA Isolation Kit (Invitrogen/Life Technologies).

RNA quality and concentration were assessed with the Agilent 2100 Bioanalyzer (Thermo Scientific). Total RNA and RNA after small RNA enrichment was analyzed using RNA 6000 Nano Kit (Agilent) and Small RNA Kit (Agilent), respectively.

### Small RNA-Sequencing, SOLiD4/SOLiD 5500xl

20 ng of EV RNA and 120 ng cell RNA, both enriched for small RNA, was used as the starting input for RNA-Seq library preparation. The preparation of the cDNA library was done according to the protocol from Applied Biosystems SOLiD™ small RNA library preparation from Invitrogen. The cDNA fragments were barcoded in the PCR amplification step to enable simultaneous sequencing of different samples in a single run. The cDNAs underwent 18 cycles of PCR using barcoded primers. The PCR products were purified using PureLink™ PCR Micro Kit (Invitrogen) and analyzed for size and concentration on Agilent 2100 Bioanalyzer using DNA HS chips. Equal molar amount of each barcoded sample were pooled together in one library, which subsequently were used in emulsion PCR to a total concentration of 0.5 pM. Approximately 1 billion enriched beads were deposited on a full glass slide for SOLiD 5500xl sequencing. Cell RNA from Hs578T and AU565 was previously sequenced on SOLiD4 [42], SRA accession SRX273660 (AU565) and SRX273750 (Hs578T). All sequencing was performed at the genomic facility, Nord University. Raw sequences were submitted to the National Center for Biotechnology Information (NCBI) Short Read Archive, Bioproject PRJNA309295

### Bioinformatics analysis

All bioinformatics analysis was performed with the CLC Genomics workbench 7.0. Raw reads were first subjected to color space adaptor trimming. Further, reads above 29 nt and below 15 nt were discarded and tags with less than 10 copies were removed. All samples were normalized by linear total count scaling, and tags with expression below 100 reads per million (RPM) in all samples were removed.

For mapping, the human genome with annotation was downloaded from the Ensembl genome database (GRCh37.74) [54]. Mapping was performed by the use of the RNA-Seq Analysis Tool in the CLC Genomics workbench, with the settings Mapping type = Also map to inter-genic regions, Color space alignment = Yes, Length fraction = 1.0, Similarity fraction = 0.9, and Strand specific = Forward.

Hierarchal clustering analyses were performed with custom setting for CLC Genomics workbench (Euclidean distance, single linkage, Log2 transformed expression values).

### Micro RNA validation assays

0.2 ng (per replicate) of small RNA enriched RNA were used for small RNA expression quantification using the miRCURY LNA™ Universal RT PCR system (Exiqon, Denmark) according to the manufacturer recommendations. All experiments were done in triplicates, and a no template control (H_2_O) was included for each primer set. The LightCycler® 96 was used for quantification, and the ΔΔCq-method was used to calculate fold change using miR-23 as internal reference. MiR-23 was validated as an internal reference by a bioinformatic approach using the SOLiD small RNA-Seq data. Here, small RNAs with a p-value (EV versus cell line) higher than 0.9 and expression above 1000 read per million (RPM) in both EV and cell lines were filtered for. This resulted in a list of seven small RNAs. MiR-23 was selected as it had the lowest standard deviation out of the miRNAs with a miRNA specific sequence identity.

## Results

### The small RNA content in breast cancer cell line-derived extracellular vesicles is distinct from their originating cells

**Fig 1:**
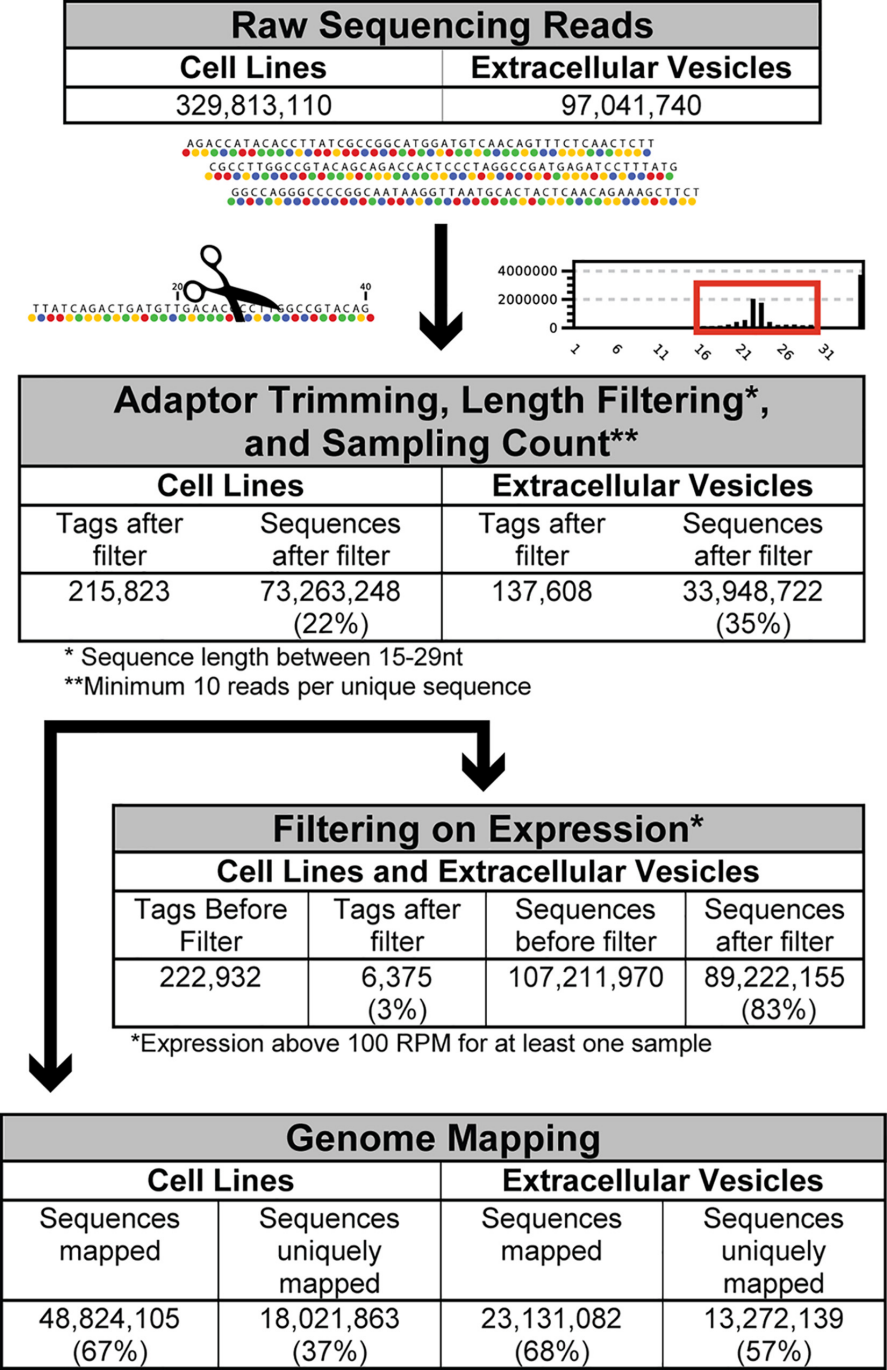
Data analysis pipeline for reads generated from small RNA SOLiD sequencing in the breast cancer cell lines and their corresponding EVs.

Cancer cells produce and release high numbers of EVs that contain a variety of biomolecules including proteins, DNA, and RNA. However, it is still debated if the secreted cargo simply reflects the intracellular content of the originating cell, or if the process is selective, allowing only certain molecules to be sorted into vesicles destined for secretion. To get a comprehensive picture of small RNA species secreted by breast cancer cells, we isolated EVs from the growth media of nine breast cancer cell lines. The cell lines were selected based on their classification as luminal (MCF7, HCC1428, and AU568), Basal B (Hs578T, MDA-MB-231, and MA-11), or Basal A (HCC1569, DU4475 and HCC1187), assumed to reflect the most common subtypes, and the heterogeneity of breast cancer. Although breast tumours are classified in six subtypes (Luminal A, Luminal B, ERBB2/HER2+, Basal-like, Claudin-low and Normal breast-like), only three different subtypes of cancer cell lines are identified based on gene expression; Luminal, Basal A and Basal B. Even though a significant discrepancy of subtypes between primary tumours and cell lines exist, comparison have shown that cell lines mirror both the genomic heterogeneity and the recurrent genome copy number abnormalities found in primary tumours. The sizes of isolated EVs ranged from 17 to 438 nm, with some variations in the relative distribution pattern among the different cell lines (data not shown). Western blot analyses confirmed that the isolated EVs from Hs578T were enriched for the tetraspanin CD63, a well-known exosomal marker protein (S1 Fig). We generated small RNA libraries from both EV and cellular fractions and sequenced them on either the SOLiD4 or SOLiD 5500xl platform (Fig 1).

Following adaptor trimming, size selection, and sampling count, 48,824,105 cellular reads and 23,131,082 EV-derived reads mapped to the human genome (GRCh37.74). Of note, whereas 57% of the reads derived from EV RNA mapped to only a single location in the genome (unique mapping), only 37% of cellular reads mapped uniquely. However, as small RNAs transcribed and processed from non-unique genomic loci might be as important as those deriving from unique loci in terms of both biological functions and cancer biomarkers, no filter was used to exclude sequences based on their mapping position. This generated a list of 222,932 tags (unique sequencing reads) for the breast cancer cell lines and their corresponding EVs. For differential expression analysis and further comparison studies, only tags with expression higher than 100 reads per million (RPM) in at least one sample were included. This resulted in a total of 6,375 tags (Fig 1; S1 Table). The sequencing data was validated by RT-qPCR (S2 Fig).

**Fig 2:**
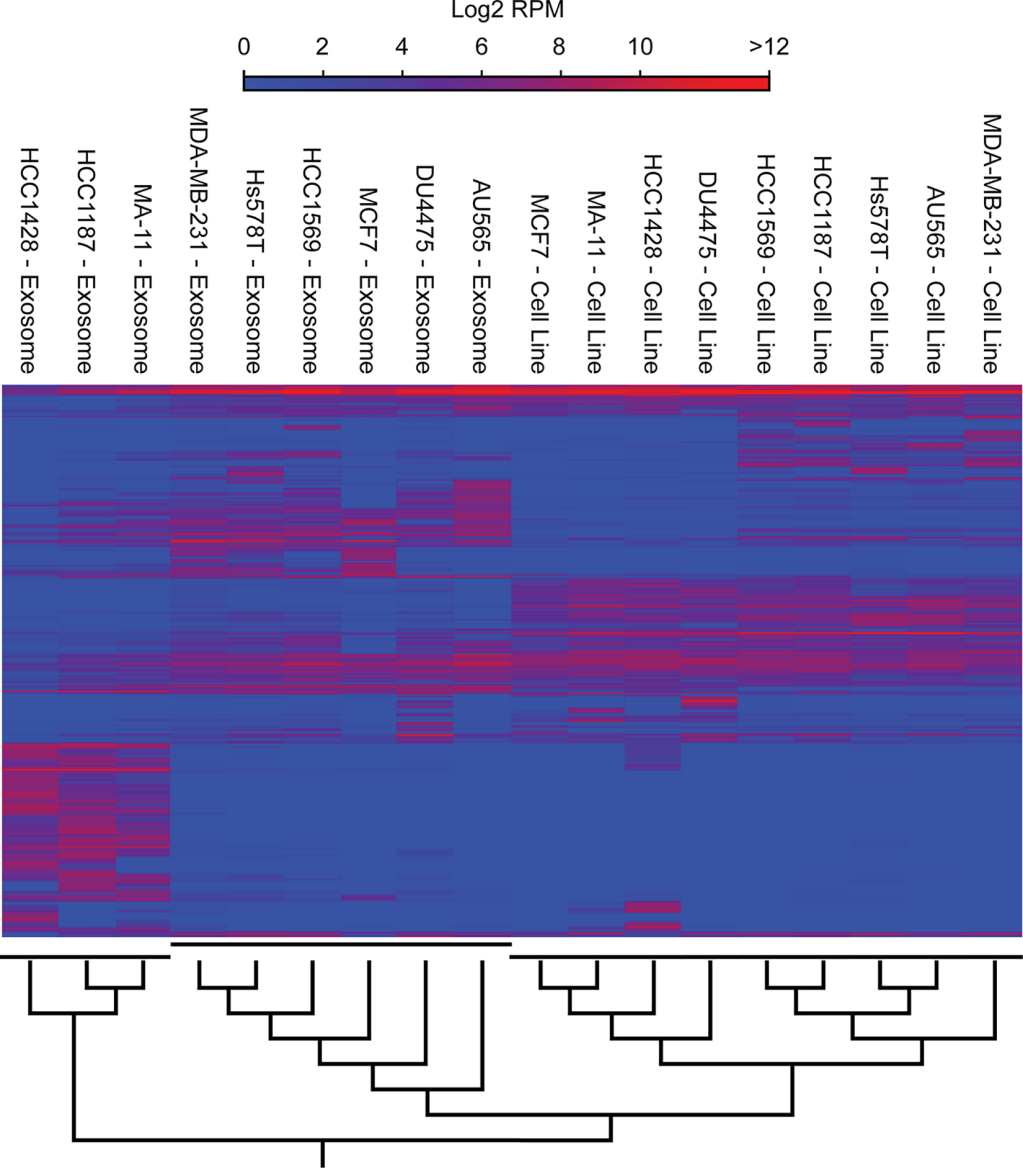
Hierarchical clustering of breast cancer cell lines and corresponding EVs on the basis of expression of 6375 unique small RNAs.

To compare the small RNA transcriptome of EVs and cells, we performed hierarchical cluster analysis (Fig 2). Based on small RNA expression, neither the breast cancer cell lines nor their corresponding EVs clustered according to their subtypes (Basal A, Basal B, or Luminal). In contrast, the intracellular small RNA content in the different breast cancer cell lines correlated more to each other, than to the small RNA content of their corresponding EVs. The small RNA content in EVs derived from three of the cell lines, MA11, HCC1428, and HCC1187, differed substantially from that of the cell lines and the rest of the EVs. This indicates that these cell lines might share common features in the sorting of small RNA species for secretion that are distinct from the other cell lines.

### The majority of small RNAs in extracellular vesicles are not micro RNAs

**Fig 3:**
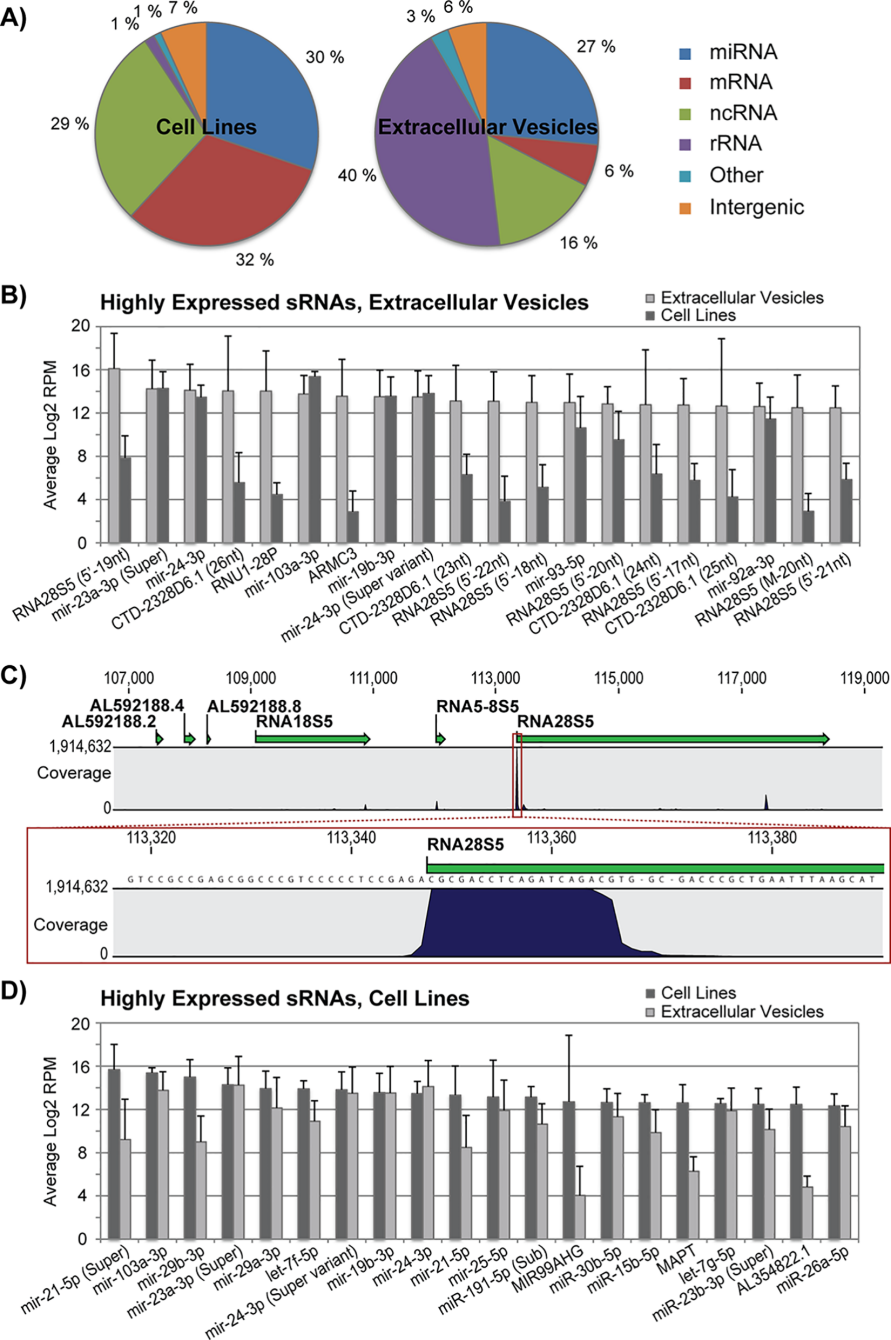
The group of non-miRNA small RNA species distinguished the EVs from their parental cell lines. **A)** Transcriptome subtype distribution of small RNA sequence reads combined for the nine breast cancer cell lines and for the nine breast cancer EVs. **B)** The expression level of the 20 most abundant small RNAs in the EVs is compared to the expression in the parental cells. RPM, reads per million. **C)** Mapping of all small RNA fragments to the 28S rRNA genomic region. Fragments compromising the first 22 nt of 28S rRNA is clearly present at a much higher level than the corresponding transcript. **D)** The expression level of the 20 most abundant small RNAs in the cell lines is compared to the expression in corresponding EVs.

As hierarchical cluster analyses clearly indicated that the small RNA content in EVs and cells differed substantially, we further investigated the origin of the small RNA species from both compartments. Approximately one third of the reads generated from the cell (27%) and EV (30%) fractions mapped to loci encoding miRNAs (Fig 3A). However, the reads were divided among a higher number of distinct miRNA species in the cellular fraction. Interestingly, EVs from the cell lines MA11, HCC1428 and HCC1187 contained fewer miRNAs species in relation to total small RNA species compared to the other EVs (Table 1). As this is not reflected in their cellular miRNA expression patterns, these cell lines appear to retain many of their miRNAs. Strikingly, a large majority of EV-derived small RNA reads (43%) mapped to rRNA (Fig 3A). In contrast, only 1% of cellular reads mapped to rRNA.

**Table 1:** Number of unique non-miRNA small RNAs and miRNAs (tags) detected in the nine breast cancer EVs.

	DU4475	HCC1569	Hs578T	AU565	MCF7	MDA-MB-231	MA-11	HCC1428	HCC1187	Average	STD
**Non-miRNA small RNA,EV**	1602 (59%)	1702 (61%)	1909 (63%)	1073 (53%)	1083 (61%)	2315 (66%)	2821 (78%)	2505 (86%)	2732 (81%)	1971 (68%)	662,6 (11%)
**miRNA,EV**	1113 (41%)	1106 (39%)	1135 (37%)	938 (47%)	679 (39%)	1182 (34%)	780 (22%)	399 (14%)	656 (19%)	888 (32%)	273,0 (11%)

To gain more insight into what makes EVs unique in their small RNA content, we examined the twenty most abundant small RNA species in the EVs (Fig 3B). Interestingly, thirteen of the most abundant small RNAs in EVs were non-miRNAs, and seven of these derived from the 28S rRNA (srRNA). Furthermore, six of these srRNAs started at the exact same position corresponding to the 5’ end of the 28S rRNA gene, but slightly differed in length (Fig 3C). RT-qPCR analyses verified a high abundance of the 19 nt 5’ srRNA in EVs compared to the cells (S3 Fig). We also examined the twenty most highly expressed small RNAs in the cell lines (Fig 3D). In contrast to the EVs, seventeen of the most abundant cellular small RNAs were miRNAs. Taken together, our analyses on the nine cell lines clearly show that the overall small RNA composition in EVs is distinct from the intracellular expression pattern.

### The cellular micro RNA expression profile is partially reflected in the extracellular vesicles

**Fig 4:**
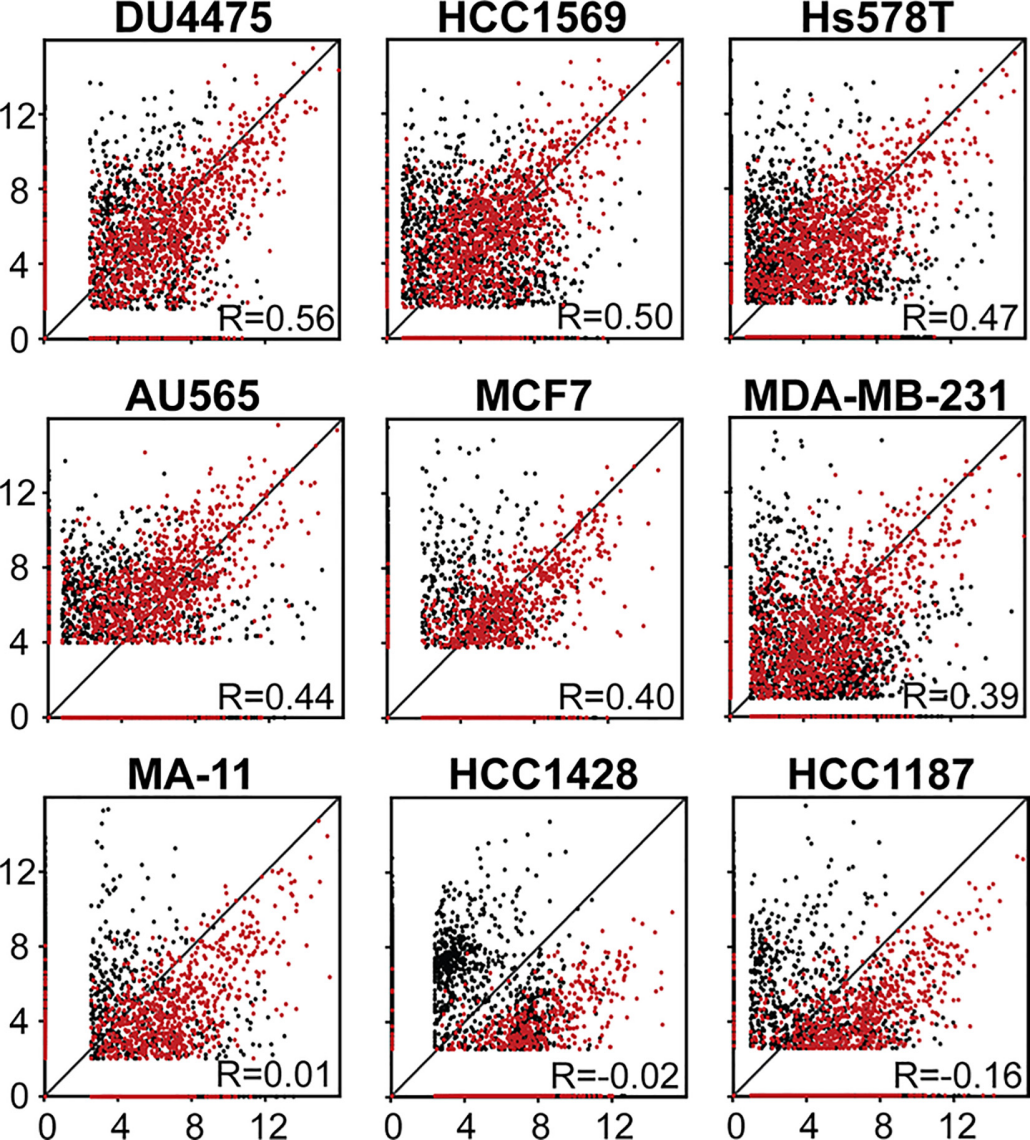
Scatterplot analysis showing the correlation between the small RNA expression pattern in nine breast cancer cell lines and the corresponding EVs isolated from cell culture media. Small RNA expression is given in log2 reads per million (RPM), with the cellular expression shown on the x-axis and the EVs on the y-axis. MiRNAs are plotted in red, while non-miRNA small RNAs are plotted in black. Pearson’s correlation is calculated for the total small RNA expression.

We then went on to analyze the correlation between all EV-derived and cellular small RNA species in each individual cell line. The cells divided into two groups based on the correlation; those that displayed moderate correlation, and those that displayed weak correlation (Fig 4). Not surprisingly, the cell lines with weak correlation are the three cell lines that clustered separately based on their EV-derived small RNA content. To study if the miRNA content in the EVs reflects the cellular miRNA expression pattern, we plotted the miRNA read counts against each other (colored red in the scatter plot) (Fig 4). Interestingly, all cell lines displayed linear correlations between EV and cellular miRNAs. Of note, this was also the case for the HCC1428, HCC1187, and MA11 cell lines even though the abundance of miRNAs was significantly higher in the cells compared to the EVs. Calculation of Pearson’s correlation coefficients confirmed a high correlation between EV-derived and cellular miRNA in all cell lines (p = 0.671 ± 0.053) (Table 2). In contrast, the correlation between EV-derived and cellular non-miRNA small RNAs was very low (p = 0.082 ± 0.221). This clearly demonstrates that whereas the cellular miRNA content is reflected in the EVs of each cell line, the composition of other small RNA species differ substantially in EVs and their corresponding originating cell line.

**Table 2:** Number of unique non-miRNA small RNAs and miRNAs (tags) detected in the nine breast cancer cell lines.

	DU4475	HCC1569	Hs578T	AU565	MCF7	MDA-MB-231	MA-11	HCC1428	HCC1187	Average	STD
**Non-miRNA small RNA, cell line**	1587 (56%)	1066 (49%)	1823 (59%)	1609 (60%)	1741 (58%)	1654 (57%)	1129 (51%)	1082 (50%)	1540 (57%)	1470 (55%)	295,8 (4%)
**miRNA, cell line**	1261 (44%)	1095 (51%)	1274 (41%)	1090 (40%)	1278 (42%)	1227 (43%)	1088 (49%)	1067 (50%)	1171 (43%)	1172 (45%)	89,0 (4%)

We observed that some miRNAs were retained in the cells, including miR-21-5p and miR-29b-3p (Fig 3D). Fifty different isoforms (isomiRs) of miR-21-5p were detected in the breast cancer cell lines (data not shown), and two isoforms including the mature sequence, were among the most highly expressed cellular miRNAs. RT-qPCR analyses confirmed higher levels of miR-21-5p in cellular compared to EV fractions (S4 Fig).

### Extracellular vesicles from breast cancer cell lines are distinct from other cancer cell lines

**Fig 5:**
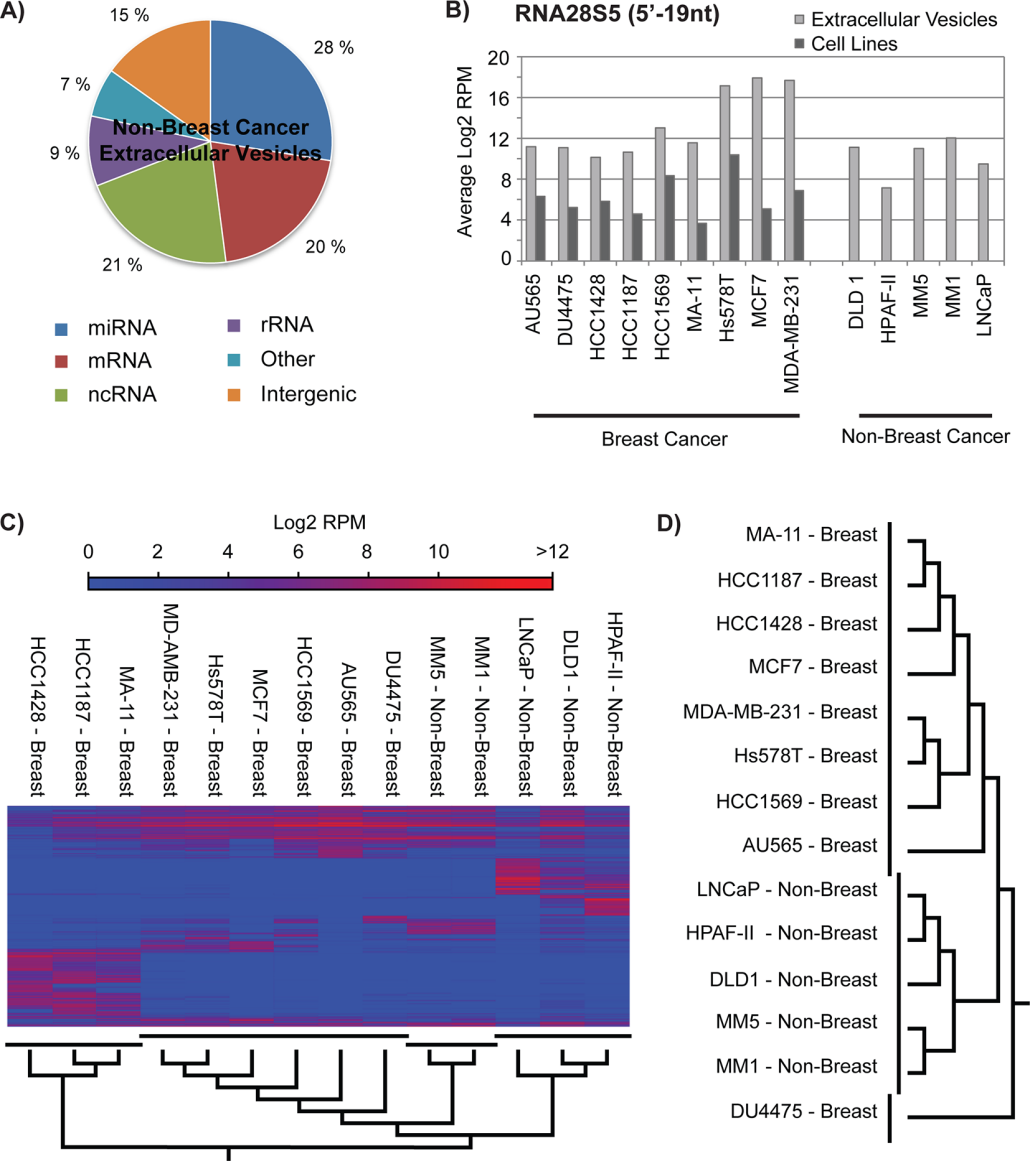
Breast cancer EVs have a different composition of small RNAs than non-breast cancer EVs. **A)** Hierarchical clustering of breast cancer EVs and non-breast cancer EVs on basis of the expression of 6569 unique small RNAs. **B)** Transcriptome subtype distribution of small RNA sequences from five non-breast cancer EVs. **C)** RNA28S5 small RNA expression in EVs from the breast cancer cells, breast cancer cells, and EVs from non-breast cancer cells. **D)** Hierarchical clustering of breast cancer EVs and non-breast cancer EVs on basis of the expression of 1087 miRNAs. **E)** Significantly differentially expressed small RNAs in breast cancer EVs as compared to non-breast cancer EVs.

To explore if the small RNA signatures of the EVs from the breast cancer cell lines are unique from other cancer EVs, we deep-sequenced the small RNA content of EVs derived from five non-breast cancer cell lines. These include LNCaP (prostate cancer), DLD1 (colorectal cancer), HPAF-II, (pancreas cancer) and two melanoma-derived cell lines (MM1and MM5). A total of 29,037,189 filtered reads were generated representing 191,210 unique small RNA sequences. Mapping of the sequencing reads to the human genome showed that 28% of the small RNA reads mapped to genes encoding miRNAs, which is similar to what was observed for the breast cancer cell lines (Fig 5A). However, only 9% of the reads mapped to rRNA genes, which is strongly opposed to the data from the breast cancer cell lines where 43% of the small RNAs derived from rRNA. Despite of this, the non-breast cancer cell lines secreted equal amounts of the 28S rRNA-derived srRNA (5’-19 nt) as the breast cancer cell lines (Fig 5B). We then combined the new sequencing tags with tags derived from the EVs of the breast cancer cell lines, using an expression cut off value of >100 RPM for at least one sample. This resulted in a list of 6,569 tags (S2 Table) that were subjected to hierarchical cluster analyses (Fig 5C). Based on the total small RNA content in the EVs, the MA11, HCC1428, and HCC1187 still formed a distinct cluster that differed from both the other breast cancer cell lines and the non-breast cancer cell lines. The rest of the breast cancer cell lines and the two melanoma cell lines formed separate clusters, indicating that the small RNA content within the EVs reflects the origin of the cancer. Interestingly, this was even more pronounced when hierarchal clustering was done based on only the miRNA composition in the EVs (Fig 5D). Here, eight of the nine breast cancer cell lines clustered together. This implies that the EV-derived miRNAs from the breast cancer cell lines form a unique signature that distinguishes them from EVs derived from other cancer types.

## Discussion

High throughput sequencing has during the last few years broadened the concept of small regulatory RNAs, and novel classes have been discovered with potential biological functions. There are several reports that miRNAs and other small RNA species are incorporated into cell-secreted vesicles, and it is now generally accepted that EVs can act as mediators of intercellular communication [12,34,55,56,57,58,59,60]. Such communication has recently been shown to play key roles in cancer progression [34,61,62,63,64,65,66]. As EVs can be detected in body fluids, their molecular content holds great premises as cancer biomarkers. Here, we find by small RNA deep sequencing that EVs derived from nine different breast cancer cell lines have a distinct composition of small RNA species compared to their originating cells. This is mostly conferred by EV-specific presence of high amounts of rRNA-derived small RNAs (srRNAs). Furthermore, we find that the miRNA content in the EVs largely reflects the intracellular miRNA expression pattern, but there are some interesting exceptions. Importantly, by comparing miRNA patterns in EVs derived from different cancer cell lines, we find that the miRNA composition generates a breast cancer-specific signature.

The small RNA content of EVs is dominated by srRNAs that appear specifically secreted from the cells. Most prominent among these are a series of srRNAs that map to the exact 5’end of the 28S rRNA, but which display size variations in their 3’ ends. In small RNA profiling studies, srRNAs have often been ignored as unspecific degradation artifacts that have been generated during for instance library preparations [49]. However, the uniform nature of the 5’ 28S rRNA fragments suggests that they are generated by precise processing of the rRNA rather than by random degradation. In line with this, a similar srRNA has been shown to associate with Ago in human fibroblasts [67]. Even though the presence of srRNAs in EVs has been reported previously [10,68], this is the first report on the specific presence of this fragment in cancer cell derived EVs. Interestingly, as high levels of the fragment was detected intracellularly in liver cells from healthy, normal mice, and that the fragment was almost completely absent in liver cells from diabetic mice [67], it is tempting to speculate that the absence could be due to specific secretion of the srRNA in the pathological condition and that this secretion has a role in the progression of the disease. This points towards a potential function of the 5’ 28S srRNAs in physiological and pathological processes. In our study, we detect the 5’ 28S rRNA fragments in EVs from all cancer cell lines analyzed. Whether this srRNA is specific for all EVs, or solely the EVs that derive from cancer cell lines, is not known, but deep sequencing analyses on exosomes derived from endothelial and immune cells failed to detect the fragment [10,69].

Switching our focus to the miRNAs, we find that miRNAs in EVs released by a particular cell line, largely reflect the miRNA expression pattern within the cells. However, for three of the cell lines very low amount of miRNAs was detected in the EVs even though the overall intracellular level of miRNAs was equal to the other cell lines. This indicates that these cell lines retain their cellular miRNAs. The molecular basis for this phenomenon is unclear, but they may lack key protein components that are required for the incorporation of miRNAs into vesicles that are destined for secretion. Even though our results show that the intracellular miRNAs are largely reflected in the EVs, we find specific miRNAs that are not equally distributed. Previous studies have indeed shown that miRNAs are not randomly incorporated into exosomes [51,70,71], and moreover, some reports have shown that exosomal miRNA expression levels are altered under different physiological conditions [34,35,72]. Here, we show that even though all breast cancer cell lines express high levels of miR-21 variants, they are not secreted in EVs. Cellular retention might thus be important for the cancerous phenotype. Increased expression of miR-21 has for a long time been associated with advanced clinical stage, lymph-node invasion, and shorter survival in breast cancer [73,74,75]. However, attempts to detect circulating miR-21 in sera from breast cancer patients have so far given inconclusive results [76,77,78,79,80,81]. Our observation that the general high expression of different miR-21 variants is not reflected in the secreted EVs, may provide an explanation for this inconsistency; miR-21 could be actively retained in breast tumors similar to the situation in breast cancer cell lines. Our data support the notion that the sorting of miRNAs into extracellular vesicles is specific and that certain miRNAs are actively retained in the cells. In line with this, a recent analysis of the miRNA profiles of EVs secreted from MCF7 and MCF10A cells confirmed different distributions of miRNA in the cell and EV profiles, including a high level of miR-21 in the cells compared to the EVs [82].

Finally, we find that the miRNA content in EVs derived from eight out of nine breast cancer cell lines generate a specific signature that make them distinct from EVs secreted by other cancer cell lines. This is an important observation that indicates that profiling of miRNAs in EVs isolated from an individual, can contribute in the diagnosis of breast cancer. Taken together, our data argue strongly that small RNA sequences are specifically produced and packaged into EVs. Recently, two papers have demonstrated that tumor-derived exosomes play key roles in the generation of pre-metastatic niches in specific organs [18,19]. Here, specific integrin molecules act as address tags for organotropic metastases [19]. In future studies, it will be interesting to evaluate if specific small RNAs associate with unique exosomes that are destined for specific metastatic niches.

## Supporting Information

S1 Fig Detection of exosomal marker CD63 in purified EVs isolated from the cell line Hs578T. Extracellular vesicles (EV), whole cell extract (WCE).
(TIF)

S2 Fig RT-qPCR validation of small RNA-Seq data. Log2 fold change expression of seven small RNAs (miR-1246, CTD-2328D6, miR-29b, miR-1260, let7f, miR-103a, miR-151a) in four cell lines (HCC1187, HCC1428, MCF7, AU565) were correlated to the small RNA-Seq data.
(TIF)

S3 Fig RT-qPCR verification of the 5’-19nt srRNA. (TIF)

S4 Fig RT-qPCR verification of miR-21. (TIF)

S1 Table Small RNA transcripts identified by small RNA sequencing of nine breast cancer cell lines and corresponding EVs. (XLSX)

S2 Table Small RNA transcripts identified by small RNA sequencing of nine breast cancer EVs and five non-breast cancer EVs. (XLSX)
