# Supplementary figures and images for: Distinct Small RNA Signatures in Extracellular Vesicles Derived from Breast Cancer Cell Lines
Source: PLoS One. 2016 Aug 31;11(8):e0161824. doi: 10.1371/journal.pone.0161824 (PMC5006963; doi:10.1371/journal.pone.0161824)

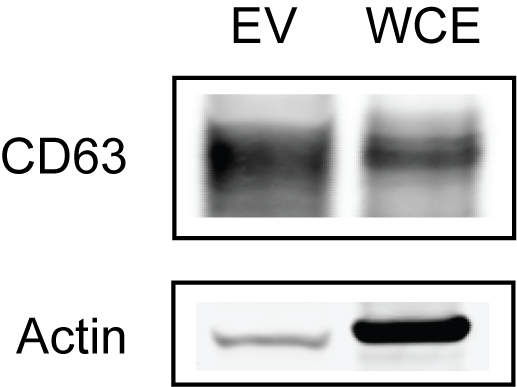

Supplement: S1 Fig — Extracellular vesicles (EV), whole cell extract (WCE). (TIF) [file pone.0161824.s001.tif]

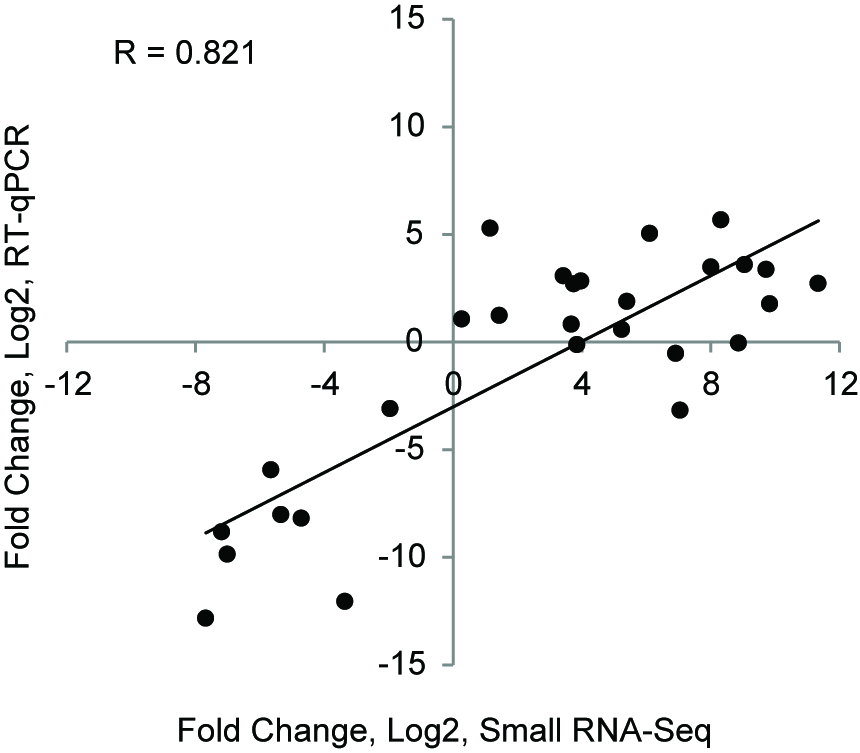

Supplement: S2 Fig — Log2 fold change expression of seven small RNAs (miR-1246, CTD-2328D6, miR-29b, miR-1260, let7f, miR-103a, miR-151a) in four cell lines (HCC1187, HCC1428, MCF7, AU565) were correlated to the small RNA-Seq data. (TIF) [file pone.0161824.s002.tif]

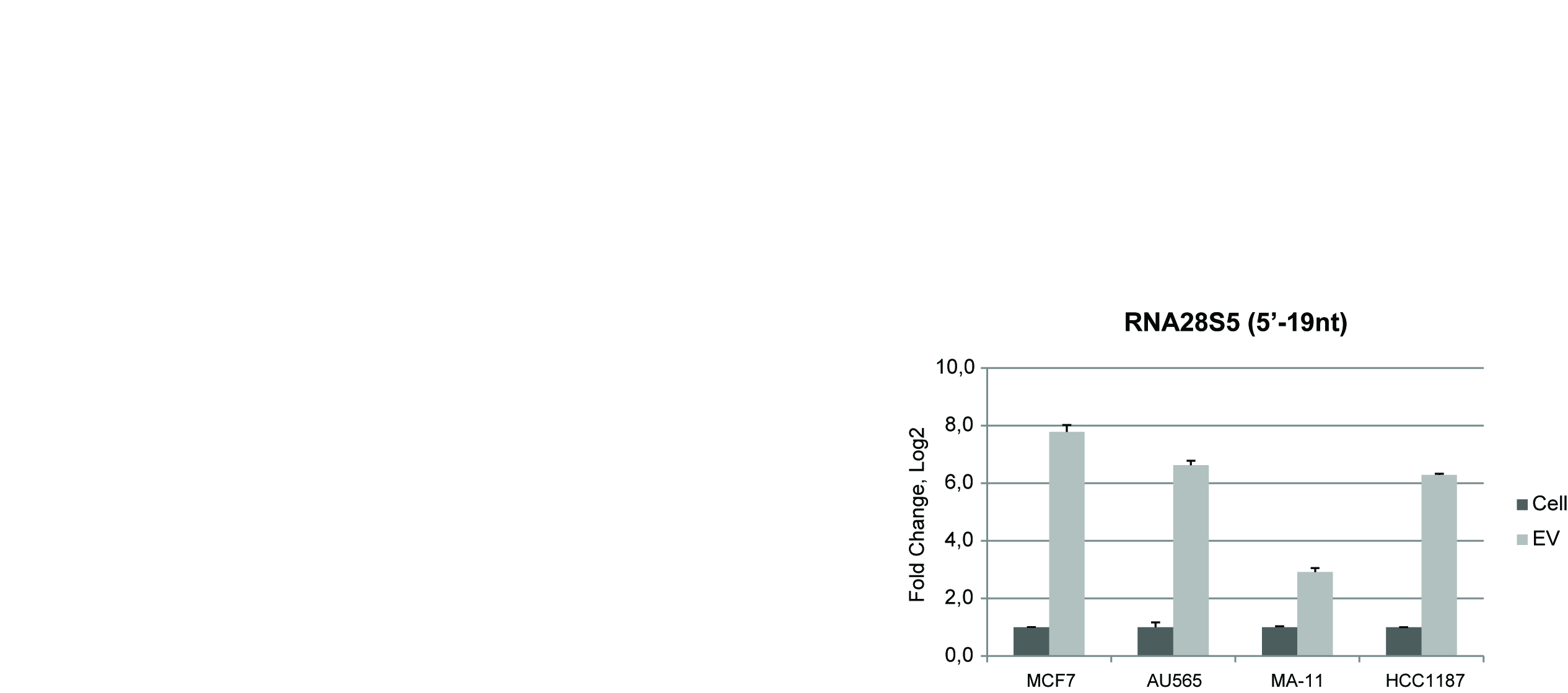

Supplement: S3 Fig — (TIF) [file pone.0161824.s003.tif]

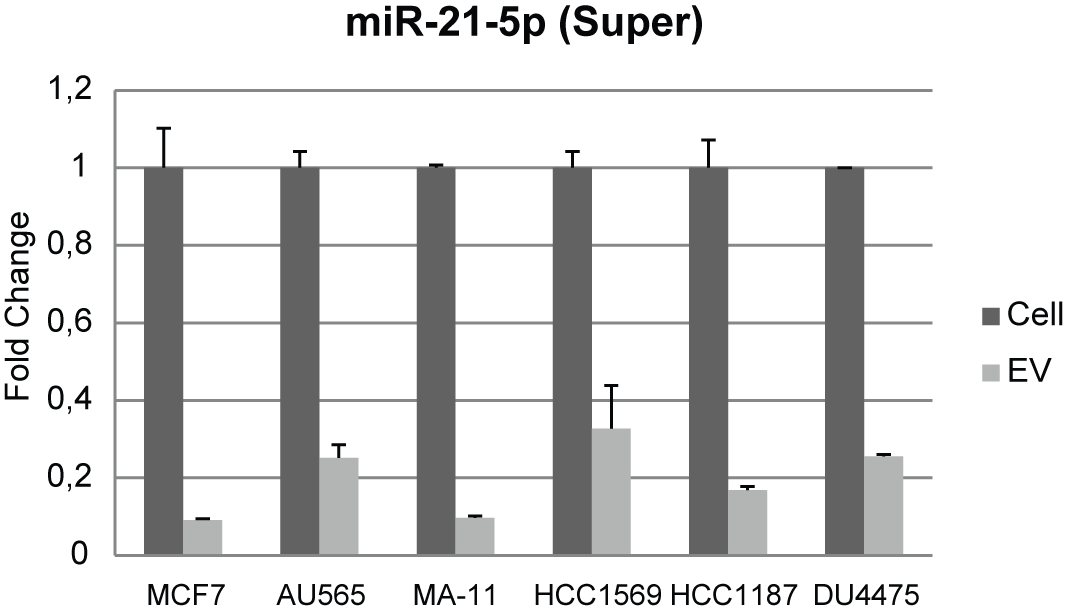

Supplement: S4 Fig — (TIF) [file pone.0161824.s004.tif]
